# Dynamic Multi-Objective Optimization in Brazier-Type Gasification and Carbonization Furnace

**DOI:** 10.3390/ma16031164

**Published:** 2023-01-30

**Authors:** Xi Zhang, Guiyun Zhang, Dong Zhang, Liping Zhang

**Affiliations:** 1 Key Laboratory of Smart Manufacturing in Energy Chemical Process, East China University of Science and Technology, Shanghai 200237, China; 2 Institute of Cotton Research, Shanxi Agricultural University, Yuncheng 044000, China; guiyunzhang@126.com (G.Z.); lipingzh2006@126.com (L.Z.); 3 Discipline of Engineering and Energy, College of Science, Health, Engineering and Education, Murdoch University, Perth, WA 6150, Australia; dongzhanghappy@126.com

**Keywords:** biochar, gasification and carbonization furnace, dynamic multi-objective optimization, Gaussian process, evolutionary algorithm

## Abstract

With the special porous structure and super-long carbon sequestration characteristic, the biochar has shown to have potential in improving soil fertility, reducing carbon emissions and increasing soil carbon sequestration. However, the biochar technology has not been applied on a large scale, due to the complex structure, long transportation distance of raw materials, and high cost. To overcome these issues, the brazier-type gasification and carbonization furnace is designed to carry out dry distillation, anaerobic carbonization and have a high carbonization rate under high-temperature conditions. To improve the operation and maintenance efficiency, we formulate the operation of the brazier-type gasification and carbonization furnace as a dynamic multi-objective optimization problem (DMOP). Firstly, we analyze the dynamic factors in the work process of the brazier-type gasification and carbonization furnace, such as the equipment capacity, the operating conditions, and the biomass treated by the furnace. Afterward, we select the biochar yield and carbon monoxide emission as the dynamic objectives and model the DMOP. Finally, we apply three dynamic multiobjective evolutionary algorithms to solve the optimization problem so as to verify the effectiveness of the dynamic optimization approach in the gasification and carbonization furnace.

## 1. Introduction

Many real-world optimization problems [1,2,3,4] have multiple conflicting objectives to be optimized. If one problem has time-varying objectives or constraints, it is called a dynamic multi-objective optimization problem (DMOP) [5]. Nowadays, there exist lots of dynamic multi-objective evolutionary algorithms (DMOEAs) [6,7,8], which have been widely applied to solve DMOPs in various areas, such as wireless sensor networks [9], financial optimization problems [10], path planning [11], and so on.

At present, the technology of crop straw returning to the field has been popularized, but the problems of pests and diseases and sowing quality caused by straw returning to the field have not been well solved [12,13]. Moreover, after straw returning to the field, biomass is degraded into carbon dioxide gas by microorganisms in a short time, which has a low carbon fixation effect and limited contribution to soil organic matter. Due to its special porous structure and super-long carbon sequestration characteristics, biochar [14] has shown its potential to improve soil fertility, reduce carbon emissions and increase soil carbon sequestration, so biochar technology will definitely replace straw-returning technology. However, the biochar technology has not been applied on a large scale to date [15,16]. The reason is that the design scale of existing gasification and carbonization equipment is too large, and the transportation radius of raw materials is large. However, agricultural wastes, such as crop straws, are low-value and difficult-to-transport materials, and the transportation cost and pretreatment cost are too high, resulting in low benefits, so it is difficult to popularize them.

The brazier-type gasification and carbonization furnace [17] belongs to the technical field of biomass comprehensive utilization equipment. It is serial equipment of a charcoal machine that carries out dry distillation, anaerobic carbonization and has a high carbonization rate in the furnace under high-temperature conditions for wood chips, rice husks, peanut shells, plant stalks, bark and other carbon-containing wood materials (the granular size is below 15 mm). The purpose of the carbonization furnace is mainly to solve the technical problems of the existing biomass carbonization equipment, such as the complex structure, long transportation distance of crop straw and other raw materials, and high cost.

In the real process of material carbonization, a certain amount of carbon monoxide, methane, oxygen and other flammable gases will be produced. In the process of recovery, purification and circulation combustion of these gases, the equipment of the brazier-type gasification and carbonization furnace will be damaged to some extent. Therefore, it is important to improve the operation and maintenance efficiency, which can not only fully provide self-sufficiency and improve the continuity of equipment and economy, but also make full use of the remaining agricultural and forestry products to turn them into waste treasure, reduce the contradiction between supply and demand of Chinese forestry resources, and contribute to environmental greening.

In this paper, we aim to solve the operation process of the brazier-type gasification and carbonization furnace as a DMOP subject to various environmental changes. Firstly, we analyze the dynamic factors in the work process of the brazier-type gasification and carbonization furnace. There probably exist environmental changes, such as the equipment capacity, the operating conditions, and the biomass treated by the furnace. Afterward, we select the biochar yield and carbon monoxide emission as the dynamic objectives, and then build the dynamic multi-objective optimization model. Next, we solve the optimization model with three DMOEAs so as to verify the effectiveness of the dynamic optimization approach in the brazier-type gasification and carbonization furnace.

The present work distinguishes with existing research mainly in two aspects. Firstly, we formulate the operation of the brazier-type gasification and carbonization furnace as a optimization problem in the presence of fairly irregular uncertainties. Secondly, most of the existing dynamic optimization algorithms are validated only on benchmark problems, and little work is applied to the real-world optimization. In this paper, three DMOEAs are examined on the real-world furnace operation problem, and the experimental results demonstrate that these algorithms can effectively track the time-varying Pareto optimal front (POF) in different environments.

Section 2 introduces the preliminary studies and related work. Section 3 elaborates the formulation of the dynamic multi-objective optimization model. Section 4 shows how to optimize the model and gives the experimental design. Section 5 concludes and discusses the future research.

## 2. Preliminary Studies and Related Work

### 2.1. Work Process of Brazier-Type Gasification and Carbonization Furnace

**Figure 1:**
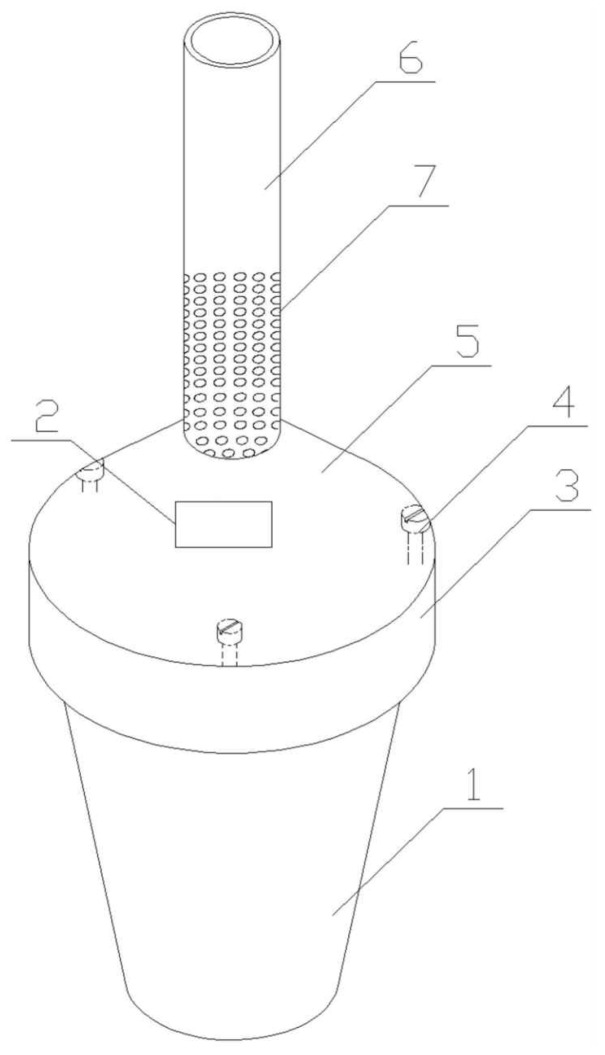
The schematic diagram of gasification and carbonization furnace: 1. furnace body; 2. ignition port; 3. fend; 4. adjusting bolt; 5. furnace cover; 6. chimney; 7. air inlet hole.

Figure 1 gives the diagram of the brazier-type gasification and carbonization furnace. As shown in Figure 1, 1 represents the furnace body, 2 represents the ignition port, 3 represents the fend, 4 represents the adjusting bolt, 5 represents the furnace cover, 6 represents the chimney, and 7 represents the air inlet hole. The furnace body is a conical cylinder with a thick upper and thin lower component, and there is no top surface or bottom surface. The furnace cover is a conical cylinder with thin upper and thick bottom component, and the bottom edge is provided with a fend. The top edge of the furnace body is uniformly arranged with a number of adjusting bolts. The furnace cover is placed on the adjusting bolt arranged at the top of the furnace body, and the protective edge covers the top of the furnace body. The chimney is arranged on the drum mouth at the top of the furnace cover, and the middle and lower parts of the chimney are uniformly provided with a number of air inlet holes.

The brazier typ- gasification and carbonization furnace is simple in structure and easy to operate. It can be used for the rapid carbonization of crop straw in the field. The working flow of the brazier-type gasification and carbonization furnace is as follows.

In the carbonization process, the carbonization furnace is first transported to the field in the appropriate position according to the size of the bottom of the furnace bricks and other bedding. Then, the bottom of the furnace body of the device is placed on the placed pad so that the furnace body is stable, and the gap formed between the pad is the first air inlet channel. Then, the biomass material to be treated is added in the furnace body, and the furnace cover is covered on the top of the furnace body so as to form a second air inlet channel between the furnace body, the furnace cover and the fend. In addition, we can adjust the distance between the furnace cover and the furnace body by adjusting bolts to control the intake air volume of the second air inlet passage. The chimney is mounted on top of the furnace cover, and the air inlet hole in the chimney can form a third air inlet.

After the carbonization furnace is installed, we ignite the biomass from the ignition port, and the biomass can start burning from top to bottom. In the process of biomass combustion, the air can be directed, respectively, from the first air inlet channel, the second air inlet channel and the third air inlet channel into the carbonization furnace in order to sustain the biomass material pyrolysis process and pyrolysis gas combustion process of oxygen demand so as to achieve rapid biomass carbonization and the full combustion of pyrolysis gas. Until the cracking is complete, the furnace cover can be taken off, buried with soil or poured with water to extinguish the output of biomass carbon—the output of biomass carbon can be applied to farmland or mixed with organic fertilizer—and the next furnace operation can continue.

### 2.2. Related Work

DMOPs [18] are characterized by the time-varying objectives, decision variables and/or constraints. We consider the minimization problem, and the DMOP is mathematically defined as

(1) $$\begin{array}{c} \min\;\;\;F\left(x,t\right)=\{f_{1}\left(x,t\right),f_{2}\left(x,t\right),\ldots ,f_{M}\left(x,t\right)\}\\ \;s.t.\;\;\;x\in [L,U] \end{array}$$

where $x=(x_{1},x_{2},\ldots ,x_{n})$ is the *n*-dimension decision variable bounded in the decision space [L,U] , where $L=(L_{1},L_{2},\ldots ,L_{n})$ , $U=(U_{1},U_{2},\ldots ,U_{n})$ , and $L_{i}$ , $U_{i}$ ∈R are the lower bound and upper bound of $x_{i}$ , respectively. *t* represents the time or environment variable. $F=(f_{1},f_{2},\ldots ,f_{M})$ denotes the set of *M* objectives to be minimized at time *t*.

To solve DMOPs, there are various kinds of DMOEAs in the literature, which can be categorized as follows: diversity approach [19,20,21], memory mechanism [22,23,24], and prediction-based method [25,26,27].

In [28], Chen et al. presented the individual diversity multi-objective optimization evolutionary algorithm (IDMOEA). IDMOEA applies a new diversity maintenance method named the individual diversity evolutionary method (IDEM) in which diversity is considered an additional objective during the optimization.

A self-organizing scout method was proposed by Branke et al. [29], and this method divides the population into two parts, that is, scouts and base population. The base population searches for optimal solutions, while the scouts are responsible for tracking the change of the optima. A fast multi-swarm optimization algorithm for DMOPs was proposed by Li et al. [30] to maintain the diversity through the search process. One parent swarm explores in the whole search space by the fast evolutionary programming algorithm whilst child swarms are generated to search for the local optima by the fast particle swarm optimization (PSO) algorithm. The multi-population strategies help with handling environment dynamisms efficiently by maintaining enough diversity and tracking the movement of multiple optima. However, too many sub-populations may slow down the search, and hence decrease the performance of optimization.

Salmond and Topcuoglu [31] presented a new hybrid strategy by integrating the memory concept with the NSGA-II algorithm (MNSGA-II) in 2016. MNSGA-II employs memory-updating mechanisms to store a number of non-dominated solutions, which can be reused in the population reinitialization for the next time. These sorts of mechanisms have been shown to be more effective on the DMOPs with periodically changing environments, i.e., the optimal solutions may return to the areas close to their previous locations, but they have the limitation that the information stored in the memory might become too redundant once changes occur.

## 3. Dynamic Multi-Objective Optimization Problem

### 3.1. Dynamic Factor Analysis

In the working process of the brazier-type gasification and carbonization furnace, it involves many dynamic factors, such as equipment capacity, operating conditions and the nature of biomass raw materials. I will give a specific analysis below:

1. The equipment capacity of the furnace is variable. In the actual working process, the wear, current, voltage, temperature and electricity of the carbonization furnace will change with the change of time, and then cause the change of equipment capacity.
2. The operating conditions of the furnace are variable. The carbonization furnace has current threshold, voltage threshold, and temperature threshold alarm settings. If the device is abnormal, the operating conditions of the device will change.
3. The biomass treated by the furnace is variable. Biomass from the carbonization furnace includes crop straw and fruit tree branches. The properties of these materials, the degree of wetness, etc., are variable.

### 3.2. Problem Formulation

The optimization process of the brazier gasification and carbonization furnace involves a number of comprehensive indexes [32], including carbon monoxide emission, particulate matter content, flue gas blackness (Ringerman blackness and grade), nitrogen oxides, biochar yield, etc. Among them, the following two performance indicators are selected as objective functions in this paper:

1. The biochar yield: The biochar yield is an indicator of the percentage of the produced biochar. The higher the biochar yield, the better the carbon fixation. The solidified carbon can be applied directly to the field or mixed with organic or chemical fertilizers, which contributes to reducing production and application processes, as well as reducing the costs.
2. Carbon monoxide emission: Carbon monoxide emission indicates whether the biomass fuel is fully burned and cracked during the combustion process. The lower the carbon monoxide emissions, the more completely the cracked gas is burned, and the less environmental pollution will be caused.

In the process of the brazier gasification and carbonization furnace, the biochar yield and carbon monoxide emission are conflicting objective functions. Therefore, we establish a dynamic multi-objective optimization model for the brazier gasification and carbonization furnace, which takes maximizing the biochar yield *B* and minimizing the carbon monoxide emission *O* as the objective function. The expression is given as follows:

(2) $$\left\{\begin{array}{c} F_{1}=\max\;\;B\\ F_{2}=\min\;\;O \end{array}\right.$$

DMOPs usually consider minimizing the objective functions. Therefore, the optimization problem is transformed into the following expression:

(3) $$\left\{\begin{array}{c} F_{1}=\min\;\;\left(-B\right)\\ F_{2}=\min\;\;O \end{array}\right.$$

**Figure 2:**
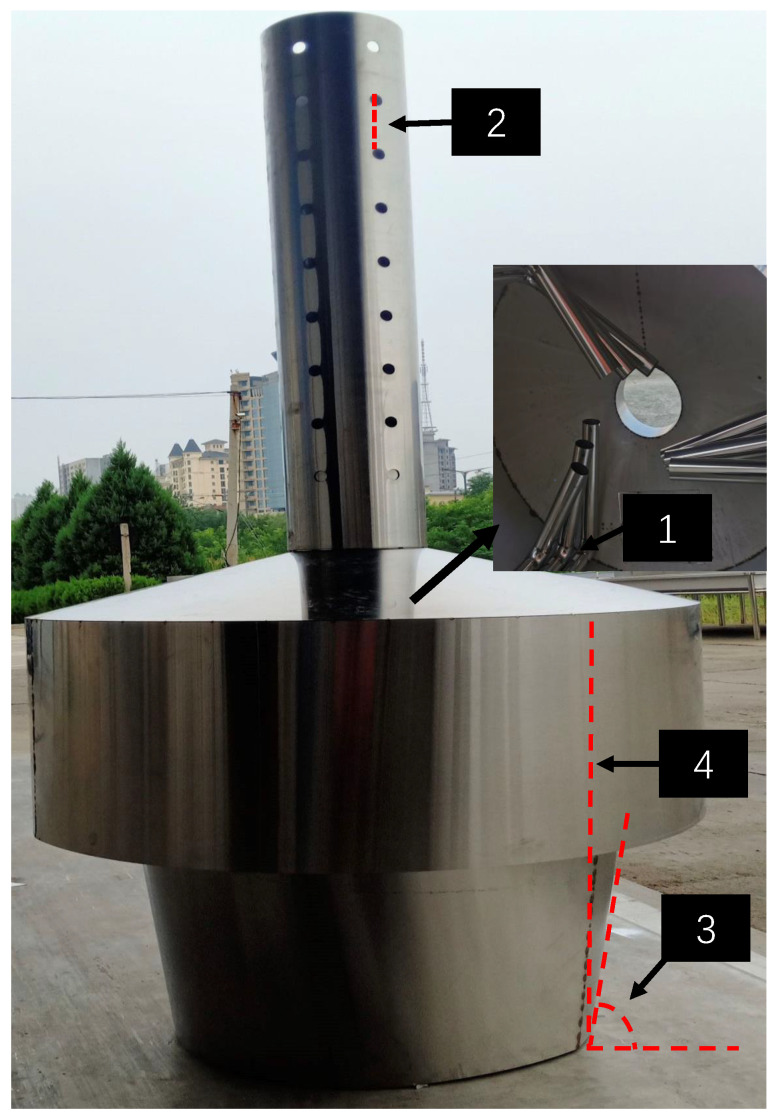
The schematic diagram of decision variables: 1. distance between the cover and the furnace body; 2. hole pitch; 3. opening angle; 4. height of the furnace body.

In addition, the decision variables involved in the optimization design of the braziertype gasification and carbonization furnace are as follows: the distance between the cover and the furnace body, the hole pitch, the opening angle and the height of the furnace body. Table 1 lists the details of decision variables in the optimization problem. To give an intuitive visualization, a schematic of decision variables is plotted in Figure 2. The distance between the cover and the furnace body can be adjusted by adjusting the nut, together with the hole pitch, which can control the oxygen content in the carbonizing furnace. This has a very important effect on whether the pyrolysis gas can be carbonized quickly and burned fully. The opening angle and height of the furnace body also determine the burning rate of biomass in the process of carbonization furnace combustion. When the oxygen content in the furnace is large, the fire will burn more vigorously, which is bound to cause a reduction in oxygen content, and it is easier to form an anaerobic environment, which is conducive to accelerating the solidification of carbon. However, when the fire is too vigorous, it can easily cause the insufficient combustion of the cracking gas. Therefore, how to balance the oxygen content in the furnace is very important for the optimization effect of the brazier-type gasification and carbonization furnace.

### 3.3. Gaussian Process

The Gaussian process (GP) [33,34] has been frequently used as a surrogate model to approximate computationally expensive fitness functions. In this paper, we apply GP modeling, which provides not only the predicted values, but also the uncertainty information of the approximate values.

The GP approximates the objective function value of an individual *x* as

(4) y(x)=μ(x)+ϵ(x)

where μ(x) denotes the mean of a regression model and ϵ(x) is a Gaussian distribution with zero mean and the standard deviation σ as

(5) $$\epsilon \left(x\right)∼N\left(0,\sigma^{2}\right)$$

The GP model is constructed by training data which are pre-evaluated individuals. Let $X={\left[x^{1},x^{2},\ldots x^{N}\right]}^{T}$ represent the training data in the decision space and $Y={\left[y^{1},y^{2},\ldots y^{N}\right]}^{T}$ as the corresponding objective vector from a multivariate Gaussian distribution, where *N* denotes the size of the training data. Afterward, let $x^{i}$ and $x^{j}$ denote two arbitrary inputs. The generally used correlation function is calculated as

(6) $$\mathrm{Corr}\left(x^{i},x^{j}\right)=\exp\left(-\sum_{k=1}^{n}\theta_{k}\left|x_{k}^{i}-x_{k}^{j}\right|\right)$$

where $\theta_{k}$ denotes the importance of this dimension, *n* is the number of decision variables, and $x_{k}$ denotes the value of the *k*th decision variable. Accordingly, for *N* samples, an N×N correlation matrix *C* is formed by

(7) $$C=\left(\begin{array}{ccc} \mathrm{Corr}(x^{1},x^{1}) & \ldots & \mathrm{Corr}(x^{1},x^{N})\\ \vdots & \ddots & \vdots \\ \mathrm{Corr}(x^{N},x^{1}) & \ldots & \mathrm{Corr}(x^{N},x^{N}) \end{array}\right)$$

For a random variable $\bar{x}$ , the posterior mean $f(\bar{x})$ and variance function $\sigma {\left(\bar{x}\right)}^{2}$ can be predicted as

(8) $$f\left(\bar{x}\right)=\hat{\mu }+r^{T}C^{-1}\left(y-1\hat{\mu }\right)$$

(9) $$\sigma {\left(\bar{x}\right)}^{2}={\hat{\sigma }}^{2}\left[1-r^{T}C^{-1}r+\frac{{\left(1-r^{T}C^{-1}r\right)}^{2}}{1^{T}C^{-1}1}\right]$$

where $r={\left(\mathrm{Corr}\left(\bar{x},x^{1}\right),\ldots ,\mathrm{Corr}\left(\bar{x},x^{N}\right)\right)}^{T}$ presents a correlation vector between $\bar{x}$ and each element $x^{i}$ in *X*. In Equations (8) and (9), the estimated mean value $\hat{\mu }$ and the estimated variance ${\hat{\sigma }}^{2}$ are obtained as follows:

(10) $$\hat{\mu }={\left(1^{T}C^{-1}1\right)}^{-1}1^{T}C^{-1}y$$

(11) $${\hat{\sigma }}^{2}=\frac{1}{N}{\left(y-1\hat{\mu }\right)}^{T}C^{-1}\left(y-1\hat{\mu }\right)$$

where 1 denotes an N×1 column vector of ones. The hyperparameters θ are obtained by maximizing the likelihood function

(12) $$\psi \left(\theta \right)=-\frac{1}{2}\left(N\ln{\hat{\sigma }}^{2}+\ln\det\left(C\right)\right)$$

where det(C) is the determinant of the correlation matrix C . After obtaining the θ values, $\hat{\mu }$ and ${\hat{\sigma }}^{2}$ can be obtained by Equations (10) and (11), respectively.

## 4. Proposed Approach

### 4.1. Dynamic Optimization Framework

**Figure 3:**
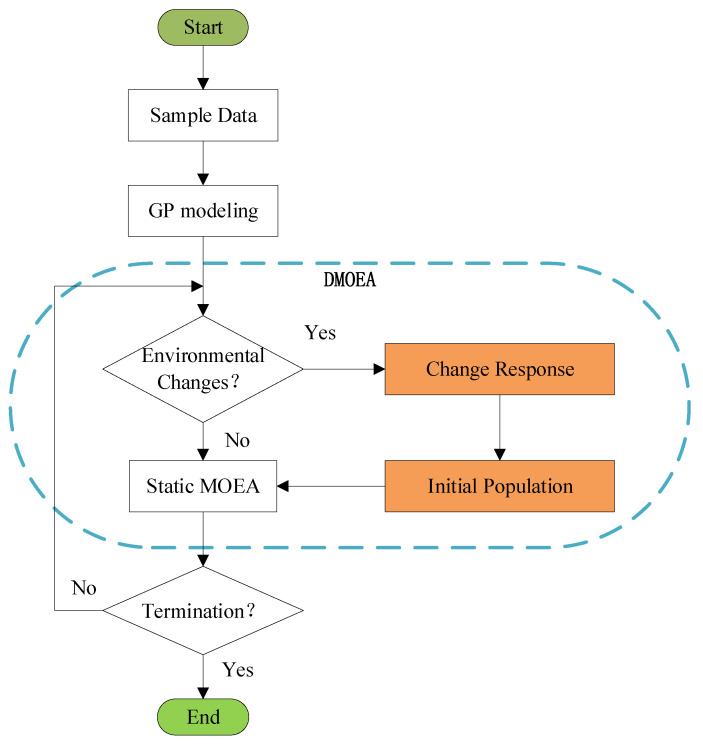
The diagram of dynamic optimization for carbonization furnace.

The main flowchart of the DMOP for the brazier-type gasification and carbonization furnace is shown in Figure 3. Firstly, we construct an optimization model of gasification carbonization furnace, in which the biochar yield and carbon monoxide emissions are served as the optimization objectives. Then, we detect the environmental changes. To be specific, 10 individuals in the decision space are selected as the detectors. If the objective values of these 10 individuals change, it indicates that the environment has changed. Next, the dynamic multi-objective evolutionary algorithm is used to optimize the optimization model of the gasification and carbonization furnace, and the optimal solutions for the new environment are obtained. Finally, if the termination conditions are reached, the whole optimization process is stopped.

### 4.2. Simulation Experiment

In this section, we adopt three classical DMOEAs to solve the DMOP for the braziertype gasification and carbonization furnace, i.e., population-prediction strategy (PPS) [35], dynamic non-dominated sorting genetic algorithm-A (DNSGA-II-A) and dynamic non-dominated sorting genetic algorithm-B (DNSGA-II-B) [36], so as to verify the effect of these algorithms in practical application. The PPS was proposed to predict a whole population rather than some isolated points for DMOPs based on a time series of center points and the previous manifold. DNSGA-II-A and DNSGA-II-B are extended from NSGA-II [37] with different change response strategies to increase the diversity of the population. DNSGA-II-A replaces a fixed percent of individuals with randomly generated solutions when changes occur, while diversity is ensured by replacing a certain part of the population with mutated solutions in DNSGA-II-B.

Because the optimization process of the brazier-type gasification and carbonization furnace is complicated, the real POF of the problem cannot be obtained, so the evaluation index related to the real POF cannot be used. Therefore, we choose the hypervolume (HV) [38] to measure the performance of the algorithms in solving the DMOP for the braziertype gasification and carbonization furnace. This metric considers both the convergence and distribution of solutions to evaluate the comprehensive quality of the resulting POF. HV calculates the hypervolume of the space enclosed by the POF and a dominated reference point ref . The larger the hypervolume value, the better the convergence and distribution of the obtained solutions. The modified HV (MHV) [39] is a modified version of HV, defined as the average of the HV values in all time steps over a run.

**Figure 4:**
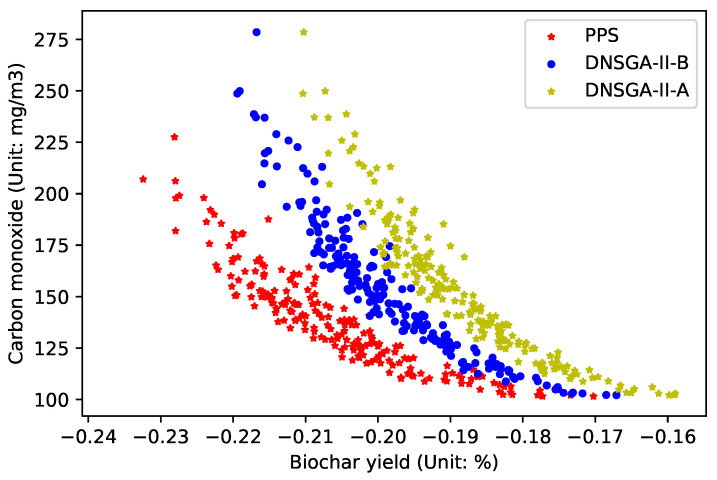
Obtained POF of three algorithms with $\tau_{t}=50$ .

Figure 4 draws the experimental results when the environmental change frequency $\tau_{t}$ is 50, that is, the environment changes once every 50 generations. This figure represents the Pareto optimal front obtained by three DMOEAs. The x-coordinate represents the objective value of the biochar yield, while the y-coordinate represents the objective value of carbon monoxide emission. As can be seen from the figure, the convergence and diversity of PPS are superior to those of DNSGA-II-A and DNSGA-II-B.

**Figure 5:**
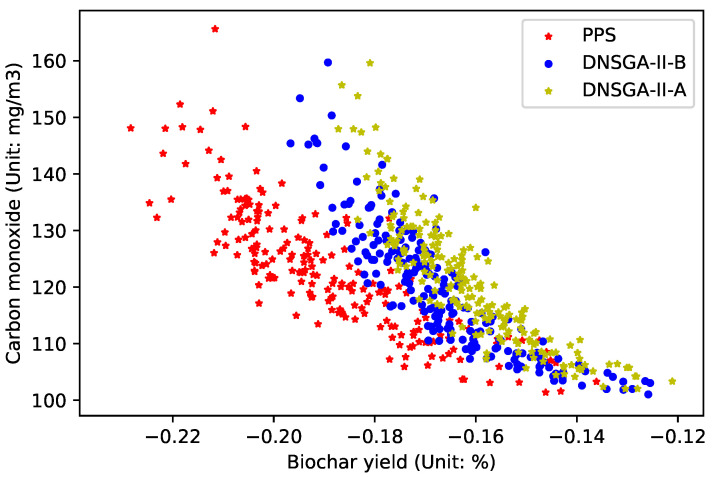
Obtained POF of three algorithms with $\tau_{t}=30$ .

**Figure 6:**
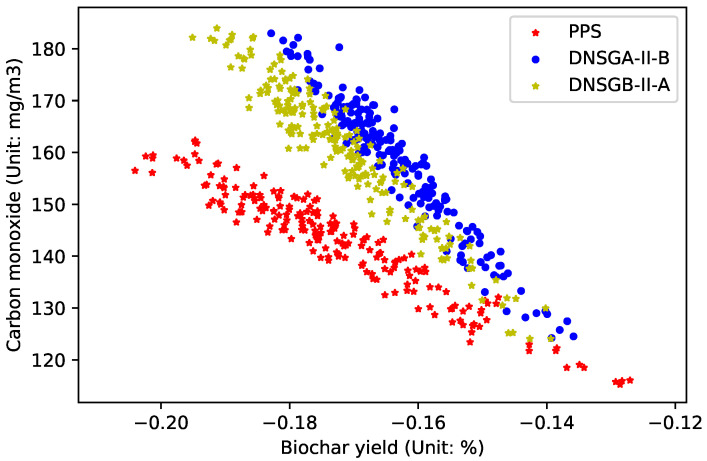
Obtained POF of three algorithms with $\tau_{t}=20$ .

**Figure 7:**
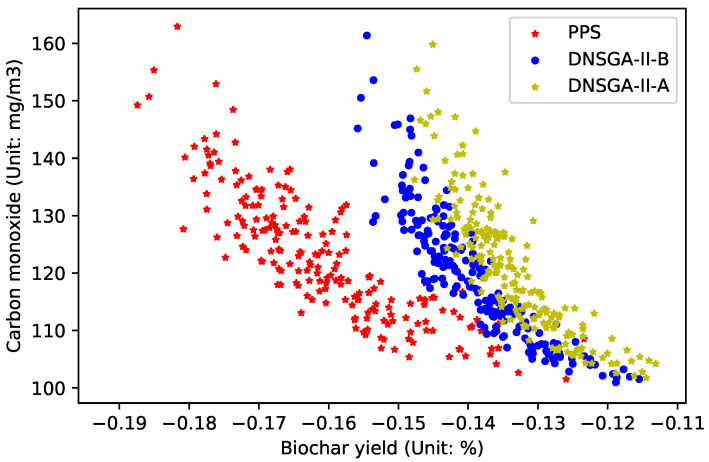
Obtained POF of three algorithms with $\tau_{t}=10$ .

In order to compare the ability of all algorithms to deal with dynamic optimization problems, Figure 5, Figure 6 and Figure 7 draw the Pareto optimal front when $\tau_{t}=30$ , $\tau_{t}=20$ and $\tau_{t}=10$ . As can be seen from the figure, when $\tau_{t}$ changes, the performance of the compared algorithms does not weaken significantly. By summarizing all experimental results, it can be concluded that compared with DNSGA-II-A and DNSGA-II-B, PPS has a better ability to solve the dynamic optimization problem of the brazier-type gasification and carbonization furnace.

**Figure 8:**
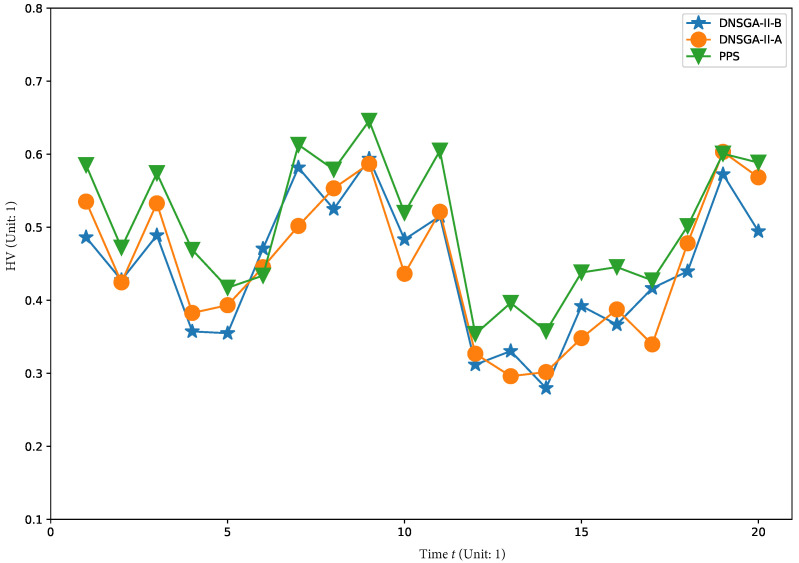
Obtained HV of three algorithms with $\tau_{t}=50$ .

**Figure 9:**
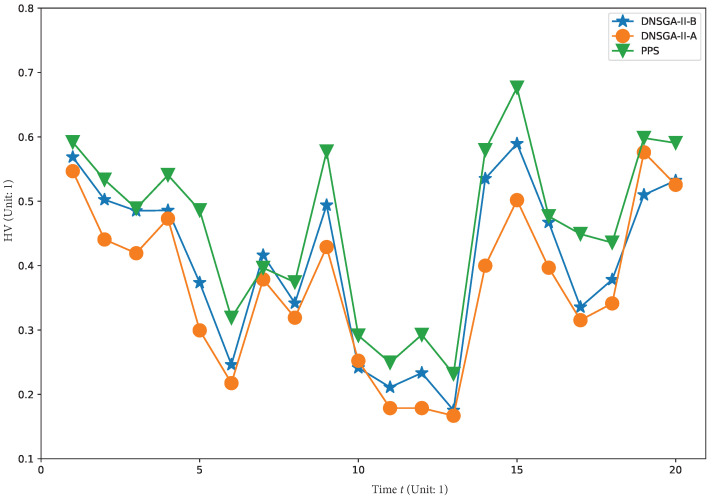
Obtained HV of three algorithms with $\tau_{t}=30$ .

**Figure 10:**
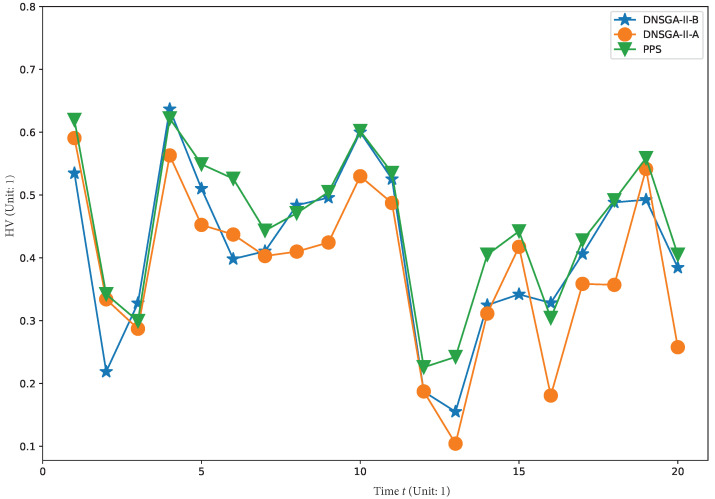
Obtained HV of three algorithms with $\tau_{t}=20$ .

**Figure 11:**
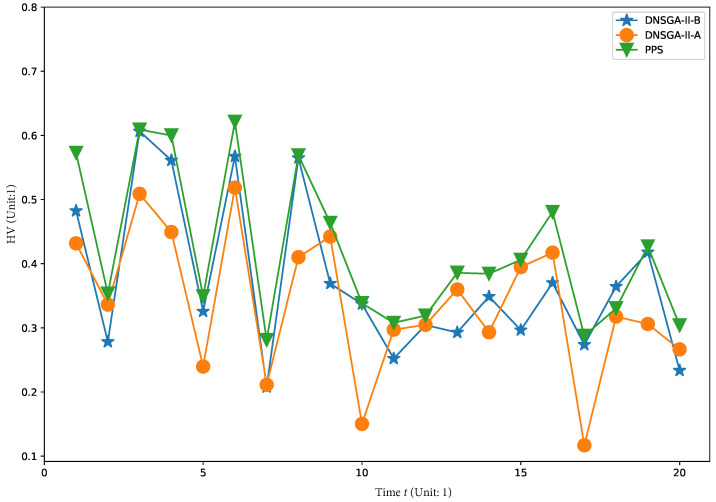
Obtained HV of three algorithms with $\tau_{t}=10$ .

To visually show the comprehensive performance of all algorithms at different environments, we plot the average HV in the first 20 changes with $\tau_{t}=50$ , $\tau_{t}=30$ , $\tau_{t}=20$ and $\tau_{t}=10$ in Figure 8, Figure 9, Figure 10 and Figure 11, respectively. It is clear to see that, compared with other algorithm, PPS achieves better HV results with time, which indicates that PPS can obtain solutions with better diversity and convergence.

**Figure 12:**
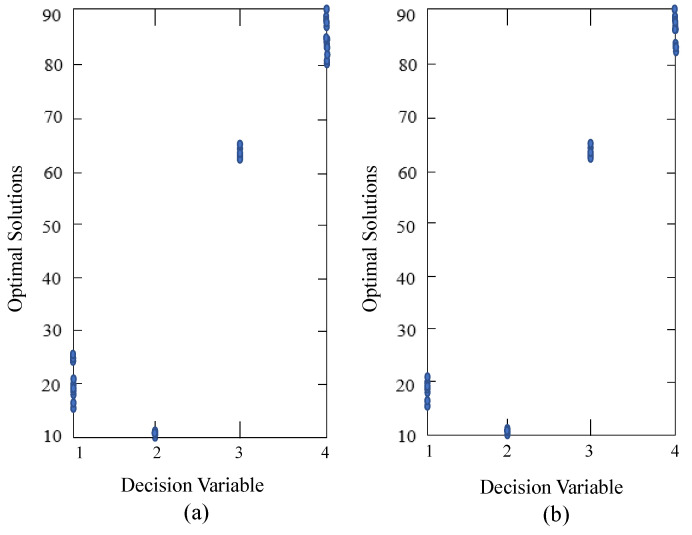
Optimal solutions obtained by PPS at (**a**) 5th generation and (**b**) 10th generation.

Figure 12 shows the optimal solutions obtained by PPS at the 5th and 10th environments with $\tau_{t}=50$ , respectively. In Figure 12, the x-coordinate represents the decision variable. Specifically, 1-4 denote the distance between the cover and the furnace body, the hole pitch, the opening angle and the height of the furnace body, respectively. The y-coordinate represents the optimal values for each decision variable.

## 5. Conclusions

In the real operation process of the brazier-type gasification and carbonization furnace, the operation efficiency may be influenced by many dynamic factors. In this paper, the operation process of the brazier-type gasification and carbonization furnace was formulated as a DMOP, aiming to improve the biochar yield and reduce the carbon monoxide emission. Additionally, three classical DMOEAs were used to solve the dynamic furnace operation problem. The experimental results demonstrate that these algorithms can effectively track the changing POF in different environments and obtain optimal solutions with good diversity and convergence.

In future, we are interested in utilizing different transfer learning methods to efficiently solve DMOPs. What is more, we will attempt to apply different DMOEAs to solve more real-life DMOPs.

## Figures and Tables

**Table 1:** The decision variables in the DMOP.

Decision Variable	Notation	Unit
Distance between the cover and the furnace body	Dis	m
Hole pitch	HP	m
Opening angle	OA	∘
Height of the furnace body	HB	m

## Data Availability

Not applicable.

## Abbreviations

The following nomenclatures are used in this manuscript:

DMOP	dynamic multi-objective optimization problem
DMOEA	dynamic multi-objective evolutionary algorithm
POF	Pareto optimal front
IDMOEA	individual diversity multi-objective optimization evolutionary algorithm
IDEM	individual diversity evolutionary method
PSO	particle swarm optimization
MNSGA-II	memory-based non-dominated sorting genetic algorithm-II
*B*	biochar yield
*O*	carbon monoxide emission
GP	Gaussian process
*y*	objective function value
μ	mean of regression model
ϵ	Gaussian distribution
*X*	training data in decision space
*Y*	objective vector
*N*	size of training data
Corr	correlation function
*C*	correlation matrix
*n*	number of decision variables
$x_{k}$	value of *k*th decision variable
*f*	posterior mean
σ	variance function
PPS	population prediction strategy
DNSGA-II-A	dynamic non-dominated sorting genetic algorithm-A
DNSGA-II-B	dynamic non-dominated sorting genetic algorithm-B
HV	hypervolume
MHV	modified hypervolume
$\tau_{t}$	change frequency
