# Correction: Zhang et al. Dynamic Multi-Objective Optimization in Brazier-Type Gasification and Carbonization Furnace. *Materials* 2023, *16*, 1164

**DOI:** 10.3390/ma17061233

**Published:** 2024-03-07

**Authors:** Xi Zhang, Guiyun Zhang, Dong Zhang, Liping Zhang

**Affiliations:** 1 Key Laboratory of Smart Manufacturing in Energy Chemical Process, East China University of Science and Technology, Shanghai 200237, China; 2 Institute of Cotton Research, Shanxi Agricultural University, Yuncheng 044000, China; 3 Discipline of Engineering and Energy, College of Science, Health, Engineering and Education, Murdoch University, Perth, WA 6150, Australia

In consideration of the contributions to this work, Feng Qian unequivocally requests the removal of his name from the author list of this publication [1]. The authorship list of this publication has been amended to reflect this, and the Author Contributions statement has been updated as follows:

**Author Contributions:** Methodology, X.Z.; resources, G.Z. and L.Z.; writing—original draft preparation, X.Z.; writing—review and editing, X.Z. and D.Z.; visualization, X.Z. and D.Z. All authors have read and agreed to the published version of the manuscript.

All authors (including Feng Qian) agreed to this change, and the Editorial Office processed this update request as per MDPI’s “Changes to Authorship” policy (https://www.mdpi.com/ethics#_bookmark3). The authors state that the scientific conclusions are unaffected. This correction was approved by the Academic Editor. The original publication has also been updated.
